# ﻿*Primulinaaureipurpurea* (Gesneriaceae), a new species from Guangxi Zhuang Autonomous Region, China

**DOI:** 10.3897/phytokeys.257.155007

**Published:** 2025-06-06

**Authors:** Bi-Dan Lai, Yi-Qin Huang, Mei-Juan Shi, Zheng-Yu Deng, Fang Wen

**Affiliations:** 1 Guangxi Vocational University of Agriculture, No. 176 east of University street, Xixiangtang District, Nanning, CN-530007, Guangxi Zhuang Autonomous Region, China Guangxi Vocational University of Agriculture Nanning China; 2 Nanning Liangqing District Dingyuan Plant Ecological Resources Monitoring Studio, No. 24 Yunzhong Street, Jinxiang Third Zone, Coastal Economic Corridor Development Zone, Liangqing District, Nanning, CN-530007, Guangxi Zhuang Autonomous Region, China Nanning Liangqing District Dingyuan Plant Ecological Resources Monitoring Studio Nanning China; 3 Guangxi Key Laboratory of Plant Conservation and Restoration Ecology in Karst Terrain, Guangxi Institute of Botany, Guangxi Zhuang Autonomous Region and Chinese Academy of Sciences, Guilin, CN-541006, Guangxi Zhuang Autonomous Region, China Guangxi Institute of Botany Guilin China; 4 Gesneriad Committee of China Wild Plant Conservation Association (GC), National Gesneriaceae Germplasm Resources Bank (NGGRB) of GXIB, Gesneriad Conservation Center of China (GCCC), Guilin Botanical Garden, Guangxi Zhuang Autonomous Region and Chinese Academy of Sciences, Guilin, CN-541006, Guangxi Zhuang Autonomous Region, China Guilin Botanical Garden Guilin China

**Keywords:** Flora of Guangxi, karst flora, new taxon, *Primulinaalbicalyx*, taxonomy

## Abstract

*Primulinaaureipurpurea*, endemic to the limestone area in Dahua County, Guangxi Zhuang Autonomous Region, China, is described and illustrated here. This species is distinguished from *P.albicalyx* by leaves sessile or with indistinct short petioles (*vs.* distinctly petiolate, 21–42 mm long in *P.albicalyx*, the following comparisons are in the same order) and blades oblanceolate, narrowly obovate to obovate (*vs.* ovate to broadly ovate); bracts smaller, lanceolate (3–4× ca. 1 mm) (*vs.* ovate to narrowly ovate, 18–25 × 9–14 mm); filaments glabrous and ca. 1.5 mm long (*vs.* sparely glandular pubescent and 2–2.5 mm long) and pistil sparsely eglandular-puberulent (*vs.* densely glandular-pubescent). According to the IUCN Red List criteria, the currently known population of this species is provisionally assessed as “Vulnerable, VU D2.”

## ﻿Introduction

The genus, *Primulina*Hance (1883), renowned for its exceptional species richness within Gesneriaceae in China, is predominantly distributed in the karst limestone regions of southern and southwestern China, with limited occurrences in northern Vietnam (Weber et al. 2011; Xu et al. 2021). This genus has long fascinated botanists due to its high levels of endemism and cryptic diversity, often exemplified by localized distribution patterns such as “one cave, one species”, “one gorge, one species”, “one hill, one species” (Monro et al. 2018; Wei 2018, 2023; Fu et al. 2022). Recent decades have witnessed a surge in taxonomic discoveries, particularly in China, where more than 220 species (with at least 215 endemics) have been documented (GRC 2025; IPNI 2025; POWO 2025; Tan et al. 2025). Morphologically, *Primulina* is characterized by rosette-like leaves, tubular-corolla shapes, and chiritoid stigmas, distinguishing it from closely related gesneriads in China (Weber et al. 2011, 2020).

This species first appeared in around 2021 on the well-known online second-hand goods shopping platform, the App, ‘Xianyu’. It attracted attention with its bright yellow corolla limb lobes, contrasting purplish-red to purple corolla tube, and moderate plant size. Upon contacting the seller, it was confirmed that the species was a wild-collected original species. However, the collector of this species consistently refused to disclose its collection location, and temporarily named it *Primulina* ‘Liudu’. Due to the lack of original location information, even though we speculated and preliminarily determined that this species was an undescribed new taxon, we were unable to process, describe, and publish it. By chance, in the second half of 2024, two authors (Lai and Huang) went to Dahua County, Guangxi, to conduct a botanical survey of karst areas, and inadvertently discovered this species that we had been longing for. After detailed comparisons between the *Primulina* ‘Liudu’ purchased online and this unknown *Primulina* species collected in the wild, we confirmed that these two plants are indeed the same species. This species is quite similar in vegetative organs to *P.linearicalyx* F.Wen, B.D.Lai & Y.G.Wei (Wen et al. 2016), but its floral organs are rather close to *P.albicalyx* B.Pan & Li H.Yang (2017), but it can be well distinguished from the above two species. We also named this species *P.aureipurpurea* F.Wen, B.D.Lai & Y.Q.Huang.

## ﻿Material and methods

In October 2024, living plants were collected during an expedition in Dahua County, Guangxi Zhuang Autonomous Region, China. These plants were subsequently introduced to the nurseries of the Gesneriad Conservation Centre of China (GCCC) and the National Gesneriaceae Germplasm Resources Bank of GXIB (NGGRB) in Guilin, Guangxi for cultivation. The flowering specimens obtained were all derived from the aforementioned wild-collected plant individuals. Cultivating the living plants in situ facilitated enhanced observation of the species’ characteristics, whilst also enabling further comparative studies with the so-called *Primulina* ‘Liudu’, previously acquired from the Chinese domestic online second-hand goods shopping platform, ‘Xianyu’. The descriptions in this study are based on observations of living plants. Given the relatively large size of the plant individuals, measurements were conducted using a ruler. Botanical morphological terminology adheres to the conventions of Beentje (2016), Harris and Harris (2001). Herbarium acronyms conform to the Index Herbariorum (Thiers 2025, continuously updated).

## ﻿Taxonomy

### ﻿ Primulina aureipurpurea

Taxon classificationPlantaeLamialesGesneriaceae

F.Wen, B.D.Lai & Y.Q.Huang sp. nov.

438A0623-4E9B-534F-B90E-BD5383723B0A

urn:lsid:ipni.org:names:77362809-1

Figs 1 , 2

#### Type.

China. • Guangxi Zhuang Autonomous Region: Dahua County, Baima Twon, Longlv village, 23.8749°N,107.7381°E, on rocks of limestone hills, elev. 490 m, 11^st^ Nov 2024 (fl.), *B.D.Lai, & Y.Q.Huang 20241109* (holotype IBK!).

#### Diagnosis.

*Primulinaaureipurpurea* is morphologically close to *P.albicalyx* in floral organ morphology (for example, corollas of similar size, corollas with yellow as the dominant color), and both are relatively geographically adjacent, but they can still be distinguished by the following combined characteristics, such as leaf sessile or with indistinct short petioles (5–10 mm long) in *P.aureipurpurea* (*vs.* distinctly petiolate, 21–42 mm long in *P.albicalyx*, the following comparisons are in the same order); blades oblanceolate, narrowly obovate to obovate (*vs.* ovate to broadly ovate); bracts lanceolate, 3–4 × ca. 1 mm in size (*vs.* ovate to narrowly ovate, 18–25 × 9–14 mm in size); filaments glabrous and ca. 1.5 mm long (*vs.* sparely glandular-pubescent and 2–2.5 mm long); pistil sparsely eglandular-puberulent (*vs.* densely glandular-pubescent).

#### Description.

Perennial herb. Rhizome gray-brown to light brown, nearly cylindrical, 2–4 cm long or longer, 7–9 mm in diameter, with indistinct nodes; lacking main root but numerous gray-brown fibrous roots, often appearing on the contact surface with soil from the bottom to the lower half of the rhizome and between nodes. Leaves 12–24, clustered at the rhizome apex, obscurely opposite to 3-verticillate, sessile or with indistinct short petioles; if petioles present, 5–10 mm long, 4–6 mm in diameter, cross-section often indistinct flattened shallow “V”-shape, densely appressed white to purplish-red pubescent (if pubescent, white under normal growth conditions and often purplish-red during drought or in low-temperature dry seasons in autumn and winter); leaf blades slightly fleshy to fleshy when fresh, thickly chartaceous when dried, adaxial surfaces green to purplish-green, often purplish-red in dry season, abaxial surface light gray-green to grayish-white accompanying slight greenish tinge, oblanceolate, narrowly obovate to obovate, 4–6 × 2.5–3.5 cm, pubescent on both surfaces as on petioles, apex obtuse to rounded, base often narrowly cuneate and gradually narrowed being petiole, margin entire; 3–4 lateral veins on each side, prominent on abaxial surface, indistinctly slightly impressed on adaxial surface. Cymes axillary, 1–4-flowered, occasionally more; peduncle purple-brown to purplish-green, 5–10 cm long, ca. 1.5 mm in diameter, densely white to purplish-red pubescent; bracts 2, opposite, light red-brown to yellow-brown, darker red-purple at apex, lanceolate, 3–4 × ca. 1 mm, margin entire, apex acute, abaxially sparsely white puberulent, adaxially glabrous; pedicel ca. 1 cm long, ca. 1 mm in diameter, densely white to purplish-red pubescent. Calyx 5-lobed to base; lobes lanceolate, ca. 5 × 1 mm, color, shape and indumentum same as on bracts. Corolla with rich colors, all lobes of the limb bright yellow, outside of corolla tube purplish-red and inside red-brown, with 3–5 deep red-brown to purplish-brown longitudinal lines along each lobe of the corolla lobes towards the inside of the corolla tube on the inner side, occasionally entirely purplish red, campanulate, ca. 3 cm long, outside sparsely puberulent and glandular-puberulent, inside nearly glabrous; corolla tube nearly cylindrical, ca. 2 mm in diameter at base, ca. 2 cm in diameter at orifice; limb distinctly bilabiate; upper lip 2-lobed to middle, lobes triangular-oblate, ca. 5 × 5.5 mm, apex rounded, margin entire; lower lip 3-lobed to middle, lobes oblong, ca. 5.5 × 4.5 mm, apex rounded, margin entire. Stamens 2, adnate to ca. 1.4 cm above the base of the corolla tube; filaments filiform, 5–7 mm long, dark purple, strongly geniculate at ca. 1/3 from base upwards, glabrous; anthers cream-white, oval, slightly constricted at the middle where filaments attach, adnate dorsally, ca. 1.5 mm long, glabrous; staminodes 3, light red-brown, lateral two distinct, filiform, apex rounded, adanate to ca. 1 cm above the base of the corolla tube, ca. 4.5 mm long, sparsely puberulent, middle one attached ca. 5 mm above the base of the corolla tube, inconspicuous, semi-transparent, ca. 0.5 mm long. Disc annular, light yellow with waxy luster, glabrous, ca. 0.5 mm high, margin entire or slightly notched. Pistil light brown to light reddish-brown, ca. 2 cm long; ovary cylindrical, ca. 1.2 cm long, ca. 1 mm in diameter, sparsely puberulent; style ca. 8 mm long, sparsely puberulent; stigma 1, upper lobe absent, lower lobe obtrapeziform, light yellow-brown, ca. 1 mm long, apex 2-lobed and lingulate. Capsule linear, ca. 3.5 cm long, glabrous.

**Figure 1.**
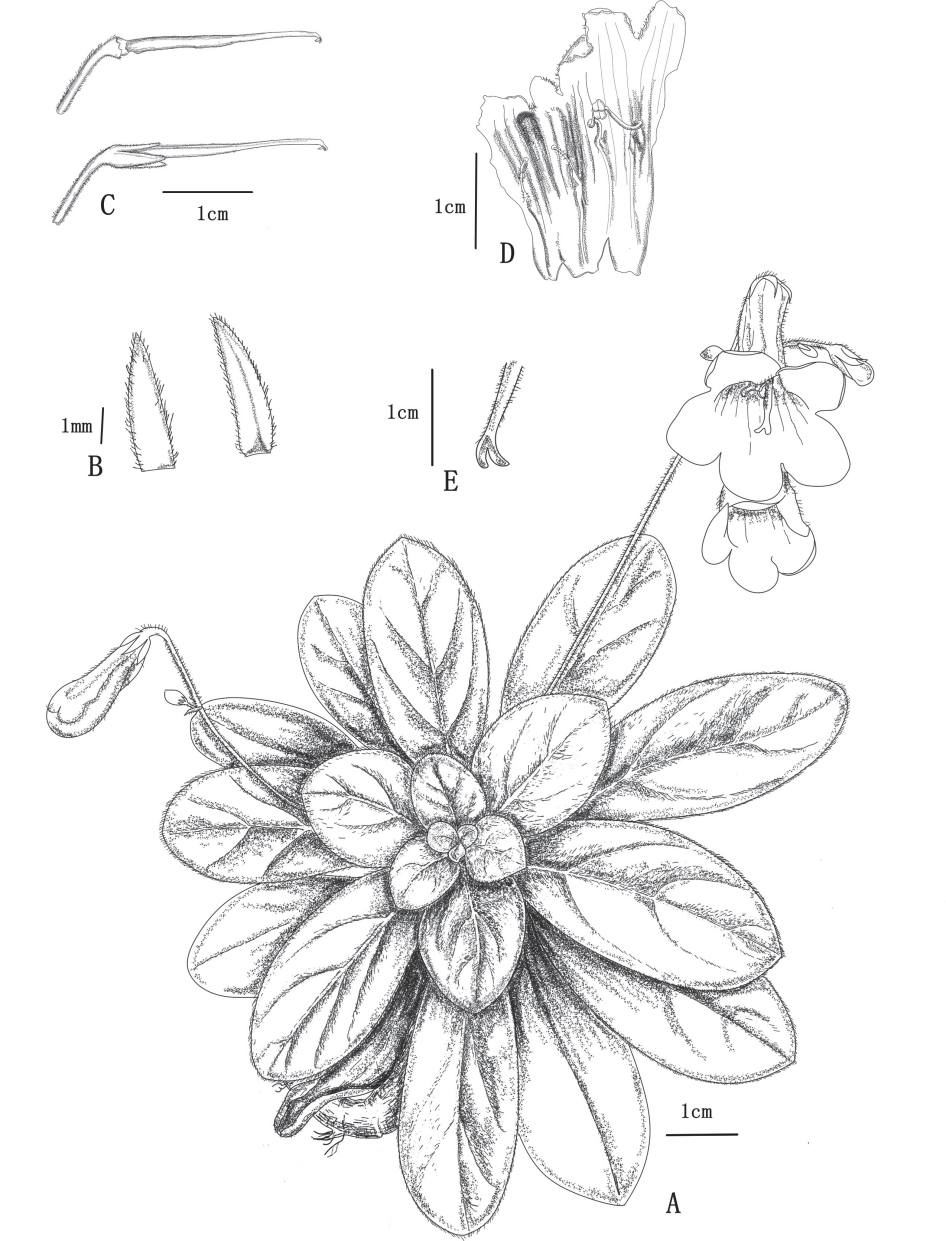
*Primulinaaureipurpurea* F.Wen, B.D.Lai & Y.Q.Huang **A** habit **B** bracts (Left: abaxial surface; Right: adaxial surface) **C** pistil (Top: pistil without calyx lobes; Bottom: pistil with calyx lobes) **D** opened corolla for showing stamens and stamindoes **E** stigma. Drawn by Bi-Dan Lai based on *B.D.Lai, & Y.Q.Huang 20241109*.

#### Distribution, habitat and provisional conservation status.

*Primulinaaureipurpurea* is currently only found in its type locality, Dahua County, Guangxi. This species prefers to grow on the upper half of steep limestone cliffs in karst hills with relatively strong sunlight. It thrives under the shelter of larger trees and shrubs directly above, or where there are overhanging rocks above, which provide overhead protection. This is likely because its leaves are fragile and easily broken, and sheltered locations effectively prevent direct rain impact that causes leaf breakage. Although this species can adapt to environments with relatively strong sunlight, its main distribution is still predominantly on north-facing hill slopes. Although it has been found on multiple limestone hills near its type locality, these limestone peaks are interconnected, so these populations on different limestone hills can be considered different sub-populations within a single population. Due to severe droughts in the type locality in the second half of 2022 and 2024, there has been a large-scale die-off of new seedlings in each sub-population, and adult plants have also experienced some mortality and growth decline. In addition, due to the variable and beautiful flower colors and strong heat and drought resistance of this species, local residents have been selling it as a wild ornamental plant, “Wild Grass from Hill,” on internet shopping platforms for several years, causing some human interference and over-collection. Currently, it has been observed that most individuals at the lower elevations of those hills have been collected, and larger groups are mainly found on limestone cliffs more than 20 meters above the ground. Furthermore, due to the limestone hills near the mountainous area where this species is distributed being mined for calcium carbonate rock (as a mining area), the type location of this species will clearly receive significant anthropogenic damage within the foreseeable decades. Therefore, considering the above factors, according to the IUCN Red List criteria, the currently known population of this species is provisionally assessed as “Vulnerable, VU D2.” Of course, with further field surveys, its endangered status may still undergo significant changes in the future (IUCN Standards and Petitions Committee 2025).

**Figure 2.**
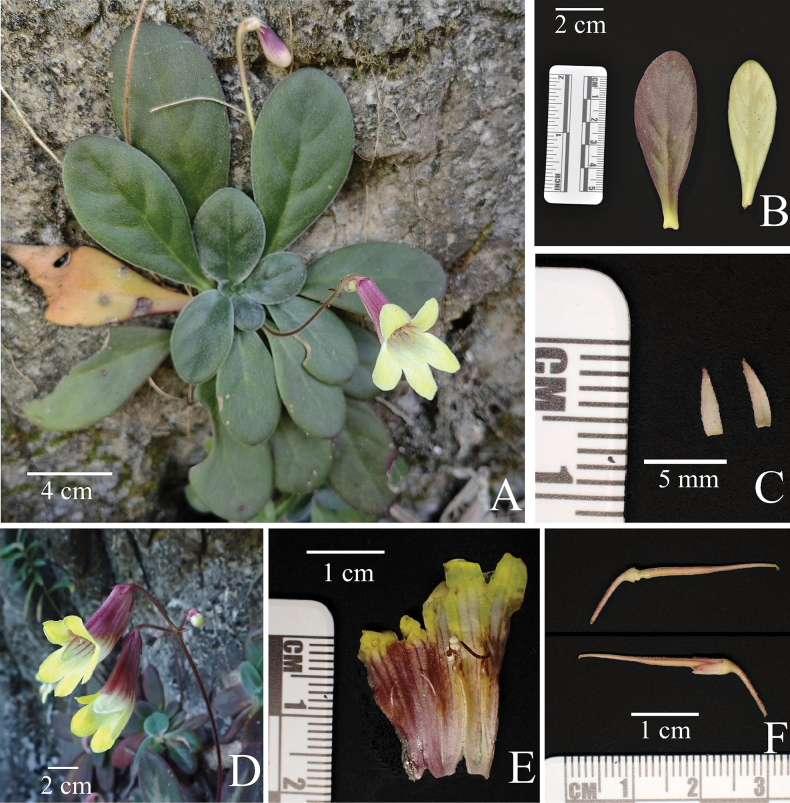
*Primulinaaureipurpurea* F.Wen, B.D.Lai & Y.Q.Huang **A** a plant in natural habitat **B** mature leaves (Left: adaxial surface; Right: abaxial surface) **C** bracts (Left: adaxial surface; Right: abaxial surface) **D** cyme with flowers, showing the lateral view of flowers **E** opened corolla for showing stamens and staminodes **F** Pistil (Top: removed calyx lobes; Bottom: kept calyx lobes). Photo by Bi-Dan Lai & Yi-Qin Huang.

#### Phenology.

Flowering period is from October to December; fruiting time is from December to January of the next year.

#### Etymology.

The specific epithet is derived from two Latin words. The prefix originates from “*aureus*”, meaning golden or gold-colored; the suffix, on the other hand, comes from “*purpureus*”, meaning purple. Therefore, it signifies the flowers of this new species exhibiting a combination of golden and purple colors. The Chinese name is given as “金玺报春苣苔” (Jin Xǐ Bào Chūn Jù Tái). The Chinese name is derived from the plant’s richly varied and dazzling corolla colors. The limb of the corolla is a bright yellow, which then transitions to a purplish-red towards the corolla tube, making it very pleasing to the eye, hence its use. On the other hand, “金玺” (Jin Xǐ) is the Chinese name for a type of gemstone within “碧玺” (Bì Xǐ, tourmaline) where yellow to amber are the primary colors. It is a borosilicate crystal containing chemical elements such as aluminum, iron, magnesium, sodium, lithium, potassium, manganese, and vanadium. Due to the presence of chromium, copper, manganese, and vanadium, it can exhibit a wide array of colors. Because of its complex composition, its colors are also complex and variable. Combining the aforementioned factors, the Chinese name “金玺” (Jin Xǐ) well embodies the highly distinctive corolla color characteristics of this new species.

## ﻿Discussion

*Primulinaaureipurpurea* is highly valued for its ornamental appeal, attributed to its charming, compact form and exquisite flowers. While it bears a close resemblance to *P.albicalyx*, a species also native to northern Guangxi Zhuang Autonomous Region, it is readily distinguishable by the diagnosis part outlined in the description. When noticing the unusual corolla color in *Primulina*, this new species also appears somewhat similar to *P.heterochroa* F.Wen & B.D.Lai (Wen et al. 2018), a species from southwestern Guangxi. However, the latter has broad elliptical leaves with distinct white veins, larger bracts, peduncles more than three times the length of those in this new species, and numerous flowers per cyme, exceeding ten, all of which are significantly different from this new species. In recent years, a number of new taxa of *Primulina* with yellow flowers have been discovered and published, such as the recently described *P.guarouba* F.Wen & W.C.Chou (Deng et al. 2024), *P.hoangmongii* K.S.Nguyen, Aver. & C.W.Lin (Nguyen et al. 2024), *P.jinyu* F.Wen, L.Ding & X.C.Ke (Ding et al. 2024), *P.nanlingensis* J.C.Luo & H.F.Chen (Luo et al. 2025), P.longzhouensisvar.flava F.Wen, W.C.Chou & Y.Z.Ge (Ge et al. 2024), *P.pseudotabacum* F.Wen & B.Pan (Mai et al. 2024b), *P.serina* F.Wen & W.C.Chou (Mai et al. 2024a), and so on. The flowering period of this new species is in October, during the second half of the year. Interestingly, considering the flowering period information of *Primulina* species, it is observed that most species with blue-purple, pink and white flowers bloom from spring to early summer, while those with yellow flowers often bloom from summer to the second half of the year. This is clearly closely related to pollinator differences and requires further experimental verification. Due to *P.aureipurpurea*’s growth in nearly bare rock locations on limestone hills, where soil attachment is minimal and winters are cold while summers are extremely hot, it exhibits high tolerance and resistance to drought, low temperatures (extreme minimum winter temperatures can reach -1 °C to 3 °C), high temperatures (extreme maximum summer rock surface temperatures can reach 43 °C), and poor soil conditions. Additionally, its compact leaves, some with variegated patterns, and its richly colored corolla make it highly ornamental, establishing it as one of the most important, if not the key, germplasm resources for ornamental breeding in *Primulina*.

## Supplementary Material

XML Treatment for Primulina aureipurpurea

## ﻿Additional information

### Author ORCIDs

Bi-Dan Lai https://orcid.org/0009-0000-3559-1283

Yi-Qin Huang https://orcid.org/0009-0006-6800-4616

Mei-Juan Shi https://orcid.org/0009-0003-3120-3863

Zheng-Yu Deng https://orcid.org/0009-0005-5527-8784

Fang Wen https://orcid.org/0000-0002-3889-8835

### Data availability

All of the data that support the findings of this study are available in the main text.

## References

[B1] Beentje H (2016) The Kew Plant Glossary, an illustrated dictionary of plant terms (2^nd^ edn). Royal Botanic Gardens, Kew.

[B2] Deng XX Xiong C Chou WC Lan DJ Huang ZC Wen F Lai BD (2024) *Primulinaguarouba*, a new endemic cave‐dwelling species of Gesneriaceae from northern Guangxi, China. Nordic Journal of Botany 2024(8): e04295. 10.1111/njb.04295

[B3] Ding L Ke XC Wen F (2024) A new lithophilous species of Gesneriaceae, *Primulinajinyu*, from the limestone area of Hubei Province, China. Taiwania 69(2): 178–184. 10.6165/tai.2024.69.178

[B4] Fu LF Monro AK Wei YG (2022) Cataloguing vascular plant diversity of karst caves in China. Biodiversity Science 30: 21537. 10.17520/biods.2021537

[B5] Ge YZ Wen F Chou WC (2024) New Taxa of Gesneriaceae from Southwest Guangxi, China—Four New Varieties of *Primulina* Hance. Botanical Research 13(5): 504–515. 10.12677/br.2024.135053

[B6] GRC (2025) Gesneriaceae Resource Centre. Internet address: https://padme.rbge.org.uk/GRC. Royal Botanic Garden Edinburgh. [Continuously updated, retrieved/accessed: 1^st^ April 2025]

[B7] Hance HF (1883) New Chinese Cyrtandreae. Journal of Botany. British and Foreign 21: 165–170.

[B8] Harris JG Harris MW (2001) Plant identification terminology: an illustrated glossary, 2^nd^ edn. Spring Lake Publishing, Payson.

[B9] IPNI (2025) International Plant Names Index. The Royal Botanic Gardens, Kew, Harvard University Herbaria & Libraries and Australian National Herbarium. [Continuously updated, retrieved: 1^st^ April 2025]. http://www.ipni.org

[B10] IUCN Standards and Petitions Committee (2025) Guidelines for Using the IUCN Red List Categories and Criteria. Version 16. Gland, Switzerland: IUCN.

[B11] Luo JC Li YQ Li YL She MZ Zeng YJ Wang FG Chen HF (2025) *Primulinananlingensis* (Gesneriaceae), a new species from the Limestone Karst of Guangdong, China. PhytoKeys 254: 99–111. 10.3897/phytokeys.254.145138 40182921 PMC11966145

[B12] Mai GD Chou WC Wen F (2024a) *Primulinaserina*, a new species of Gesneriaceae from northern Guangxi, China. Gardens’ Bulletin (Singapore) 76(2): 285–291. 10.26492/gbs76(2).2024-11

[B13] Mai GD Pan B Wen F (2024b) *Primulinapseudotabacum* (Gesneriaceae), a new species from Guangxi Province, China. Annales Botanici Fennici 61(1): 141–146. 10.5735/085.061.0121

[B14] Monro AK. Bystriakova N Fu LF Wen F Wei YG (2018) Discovery of a diverse cave flora in China. PLoS ONE 13(2): e0190801. 10.1371/journal.pone.0190801 PMC5802439 29415039

[B15] Nguyen KS Averyanov LV Lin CW (2024) *Primulinahoangmongii* (Gesneriaceae), a new species from northern Vietnam. Phytotaxa 645(2): 179–185. 10.11646/phytotaxa.645.2.7

[B16] POWO (2025) Plants of the World Online. Facilitated by the Royal Botanic Gardens, Kew. http://www.plantsoftheworldonline.org/ [Continuously updated, accessed: 1^st^ April 2025]

[B17] Tan K Ning Y Wang RF Wang Q Liang DP Xin ZB Wen F (2025) A dataset on the checklist and geographical distribution of Gesneriaceae in China. Shengwu Duoyangxing 33: 23275. 10.17520/biods.2023275

[B18] Thiers B (2025) Index Herbariorum: A global directory of public herbaria and associated staff. New York Botanical Garden’s Virtual Herbarium. http://sweetgum.nybg.org/science/ih/ [Continuously updated, accessed: 1^st^ April 2025]

[B19] Weber A Middleton DJ Forrest A Kiew R Lim CL Rafidah AR Sontag S Triboun P Wei YG Yao TL Möller M (2011) Molecular systematics and remodeling of *Chirita* and associated genera (Gesneriaceae). Taxon 60(3): 767–790. 10.1002/tax.603012

[B20] Weber A Middleton DJ Clark JL Möller M. (2020) Keys to the infrafamilial taxa and genera of Gesneriaceae. Rheedea—Special Gesneriaceae issue 30(1): 5–47. 10.22244/rheedea.2020.30.01.02

[B21] Wei YG (2018) The Distribution and Conservation Status of Native Plants in Guangxi, China. China Forestry Publishing House, Beijing.

[B22] Wei YG (2023) Catalogue and Red List of Plant Species in Guangxi. China Forestry Publishing House, Beijing.

[B23] Wen F Lai BD Zhao ZG He JY Jiang BS (2015) *Primulinaheterochroa* (Gesneriaceae), a new species from a tropical limestone area in Guangxi, China. Willdenowia 45(1): 45–51. 10.3372/wi.45.45104

[B24] Wen F Lai BD Zhao ZG Wang BM Wei YG (2016) *Primulinalinearicalyx* (Gesneriaceae), a new species from Guangxi, China. Phytotaxa 269(1): 041–046. 10.11646/phytotaxa.269.1.5

[B25] Xu MZ Yang LH Kong HH Wen F Kang M (2021) Congruent spatial patterns of species richness and phylogenetic diversity in karst flora: Case study of *Primulina* (Gesneriaceae). Journal of Systematics and Evolution 59(2): 251–261. 10.1111/jse.12558

[B26] Yang LH Pan B (2017) *Primulinaalbicalyx* (Gesneriaceae), a new species from a karst area in Guangxi, China. Willdenowia 47: 311–316. 10.3372/wi.47.47312

